# NLRP3 inflammasome pathway activation is associated with cardiac remodeling, gut microbiota dysbiosis, and dietary protein restriction in a rat heart failure model: an integrative exploratory analysis

**DOI:** 10.3389/fgene.2026.1852819

**Published:** 2026-08-18

**Authors:** Xiaopeng Li, Chen Yang, Mengmeng Nie, Yong Zhang

**Affiliations:** Department of Cardiology, The First Affiliated Hospital of Anhui Medical University, Hefei, Anhui, China

**Keywords:** NLRP3 inflammasome, heart failure, cardiac remodeling, gut microbiota, 16S rRNA, dietary protein restriction, probiotics, trimethylamine N-oxide

## Abstract

**Objective:**

To characterize protein expression of NLRP3 inflammasome pathway components (NLRP3, TXNIP, ASC, IL-1β, IL-18, Caspase-1) in failing myocardium alongside exploratory mRNA profiling, elucidate their correlation with gut microbiota 16S rRNA gene profiles and the metabolite trimethylamine N-oxide (TMAO), and evaluate how dietary protein restriction and probiotic intervention modulate these pathways to influence heart failure progression.

**Methods:**

Five groups of male SD rats were established: Control, HF, HF + Pro, LHF, and LHF + Pro, using abdominal aortic constriction. Comprehensive genomic and molecular analyses included: (1) quantitative real-time PCR (qRT-PCR) profiling of six key inflammasome genes (NLRP3, TXNIP, ASC, IL-1β, IL-18, Caspase-1); (2) Western blot protein quantification of the NLRP3 pathway; (3) gut microbiota 16S rRNA gene sequencing (V3-V4 region, Illumina NovaSeq); (4) serum biomarker enzyme-linked immunosorbent assay (IL-18, IL-1β, TNF-α, brain natriuretic peptide, TMAO); and (5) histopathological and ultrastructural analysis of myocardial tissue.

**Results:**

Serum IL-18 protein levels were significantly elevated in the LHF group compared to controls (P < 0.05) by ELISA, with the most pronounced increase observed under dietary protein restriction; however, IL-18 mRNA expression by qRT-PCR showed directionally consistent but statistically non-significant trends across groups (P = 0.929, n = 3 per group). TMAO levels were decreased in HF and HF + Pro groups but paradoxically elevated in the LHF group, a finding discussed in detail in the context of dietary substrate availability and hepatic FMO3 activity. Western blot results showed that the expression of NLRP3, TXNIP, ASC, IL-1β, and IL-18 proteins increased in all heart failure groups compared to the control group, with the most significant increase in the low-protein diet group. Pathological examination revealed increased myocardial fibrosis in heart failure groups, which was further aggravated by low-protein diet, while probiotic intervention partially improved these pathological changes.

**Conclusion:**

Heart failure is associated with upregulation of NLRP3 inflammasome proteins (NLRP3-TXNIP-ASC-IL-1β/IL-18 axis) alongside gut microbiota dysbiosis; mRNA-level trends were directionally consistent but did not reach statistical significance. Low-protein diet markedly amplifies inflammasome protein expression and cardiac remodeling, while probiotic supplementation is associated with reduced NLRP3 pathway protein expression through microbiome restoration. These findings suggest NLRP3-related protein signatures as candidate biomarkers and potential therapeutic targets in heart failure, warranting further mechanistic investigation.

## 1 Introduction

Heart failure (HF) represents the end stage of various cardiac diseases, characterized primarily by decreased cardiac pumping function and inability to meet the body’s metabolic demands. According to the Global Burden of Disease study, age-standardized mortality rates related to heart failure increased by 38.3% between 1990 and 2017, with approximately 3 million deaths worldwide in 2019 (Roth et al., 2020). This growing disease burden is closely related to population aging, improved survival from coronary heart disease, and rising prevalence of metabolic diseases. Despite significant advances in heart failure treatment over recent decades, rehospitalization and mortality rates remain high, urgently requiring the identification of new therapeutic targets (Savarese and Lund, 2017).

The pathophysiological mechanisms of heart failure are complex, involving hemodynamic abnormalities, ventricular remodeling, neuroendocrine system activation, myocardial fibrosis, and inflammatory responses (Murphy et al., 2020). Recent studies have found that chronic low-grade inflammation plays a central role in the development and progression of heart failure, with inflammatory factors such as interleukin-1β (IL-1β), interleukin-18 (IL-18), and tumor necrosis factor-α (TNF-α) not only participating in myocardial injury but also mediating ventricular remodeling and cardiac function deterioration (Van Tassell et al., 2014; Abbate et al., 2020).

The NLRP3 (NOD-like receptor family pyrin domain containing 3) inflammasome is an important component of the innate immune system. As an intracellular pattern recognition receptor, it can sense pathogen-associated molecular patterns (PAMPs) and damage-associated molecular patterns (DAMPs), thereby initiating downstream inflammatory cascade reactions (Toldo and Abbate, 2018). The NLRP3 inflammasome consists of NLRP3 protein, apoptosis-associated speck-like protein (ASC), and cysteine protease-1 (Caspase-1). Upon activation, it promotes the maturation and secretion of IL-1β and IL-18, causing tissue inflammatory injury (Swanson et al., 2019; Kelley et al., 2019).

Multiple studies have shown that the NLRP3 inflammasome plays a key role in cardiovascular diseases. In myocardial ischemia/reperfusion injury, NLRP3 expression is significantly upregulated, mediating myocardial damage and adverse remodeling (Sandanger et al., 2013). Kawaguchi et al. first reported the role of inflammasome in myocardial ischemia/reperfusion injury, finding that ASC gene knockout mice had reduced myocardial infarct size and attenuated ventricular remodeling (Kawaguchi et al., 2011). In pressure overload-induced heart failure models, NLRP3 gene knockout significantly reduces IL-1β production and alleviates myocardial hypertrophy and contractile dysfunction (Bracey et al., 2013). MCC950, as a selective NLRP3 inhibitor, has been proven to improve myocardial remodeling and enhance cardiac function (van Hout et al., 2017).

Thioredoxin-interacting protein (TXNIP) is a key molecular switch linking oxidative stress with inflammatory responses (Zhou et al., 2010). Under normal conditions, TXNIP binds to thioredoxin (TRX), keeping the NLRP3 inflammasome in an inactive state. When oxidative stress occurs, reactive oxygen species (ROS) production causes TXNIP to dissociate from TRX, and free TXNIP then binds to NLRP3, initiating inflammasome assembly and activation (Lane et al., 2013; Mohamed et al., 2014). This mechanism is particularly important in myocardial ischemia/reperfusion injury. Liu et al. found enhanced interaction between TXNIP and NLRP3 after myocardial ischemia/reperfusion, and inhibition of the TXNIP-NLRP3 axis significantly reduced myocardial injury (Li et al., 2014).

The gut microbiota is closely related to host health and is referred to as the body’s ‘second genome’. The human gut harbors approximately 1,000-1,150 microbial species, with a total genome far exceeding the human genome itself (Qin et al., 2010; Witkowski et al., 2020). The gut microbiota affects cardiovascular health through multiple pathways including production of metabolites, regulation of immune responses, and maintenance of intestinal barrier function. Microbial metabolism of dietary phosphatidylcholine generates trimethylamine N-oxide (TMAO), a gut-derived metabolite associated with cardiovascular risk (Wang et al., 2011; Tang et al., 2013). This bidirectional regulatory relationship between the gut and heart is termed the ‘gut-heart axis’ (Tang et al., 2017; Cui et al., 2018; Pasini et al., 2016).

Studies have shown that heart failure patients exhibit gut microbiota dysbiosis, characterized by decreased microbiota diversity, reduced butyrate-producing bacteria, and increased pathogenic bacteria (Sandek et al., 2007; Krack et al., 2005). In heart failure, decreased cardiac output leads to insufficient visceral perfusion and intestinal wall ischemic edema, thereby disrupting intestinal barrier function and causing bacterial and endotoxin translocation, triggering systemic inflammatory responses (Sandek et al., 2014). This mechanism has been conceptualized as the ‘gut hypothesis of heart failure’, providing new intervention targets for heart failure treatment (Krack et al., 2005).

In summary, NLRP3 inflammasome activation, gut microbiota dysbiosis, and malnutrition all participate in the development and progression of heart failure, but the interrelationships and specific regulatory mechanisms remain unclear. This study aims to: (1) investigate the correlation between NLRP3 inflammasome pathway protein expression and gut microbiota metabolite TMAO in heart failure rats; (2) observe the effects of low-protein diet on heart failure progression and NLRP3 pathway activation; (3) evaluate the protective effects and possible mechanisms of probiotic intervention on heart failure. This study is expected to provide new experimental evidence for nutritional intervention and microecological treatment of heart failure, laying the foundation for clinical translation of the ‘gut-heart axis’ theory.

## 2 Materials and methods

### 2.1 Experimental animals and grouping

Healthy male SD rats weighing 200–250g (SPF grade) were purchased from the experimental animal center. All animals were housed at 22 °C ± 2 °C with 50% ± 10% humidity, 12 h/12 h light/dark cycle, with free access to water and food. After 1 week of adaptive feeding, rats were randomly divided into 5 groups using a random number table: Group A (LHF): heart failure + low-protein diet (n = 4), Group B (LHF + Pro): heart failure + low-protein diet + probiotics (n = 6), Group C (HF + Pro): heart failure + normal diet + probiotics (n = 5), Group D (HF): heart failure + normal diet (n = 5), Group E (Control): normal control group (n = 6). This study was approved by the Medical Ethics Committee of Feidong County People’s Hospital (Approval No. dw2024002). All animal procedures were conducted in accordance with the approved protocol and institutional requirements.

### 2.2 Heart failure model establishment and intervention methods

A pressure overload heart failure model was established using abdominal aorta constriction. After rats were anesthetized with 2% pentobarbital sodium (40 mg/kg) via intraperitoneal injection and fixed in supine position, a ∼2 cm midline incision was made, and the abdominal wall muscle layer was bluntly dissected to open the abdominal cavity. The abdominal aorta was carefully freed, and approximately 0.5 cm above the renal arteries, the abdominal aorta was ligated together with a 7-gauge needle using 4–0 silk thread. After ligation, the needle was removed, creating approximately 60%-70% vascular stenosis. The abdominal wall was sutured layer by layer, and penicillin was injected intramuscularly postoperatively to prevent infection. The control group only exposed the abdominal aorta without ligation. Postoperative general condition was observed, including mental status, activity ability, respiratory rate, and fur luster. Animals that died within 24 h post-surgery were excluded. In total, 35 rats underwent abdominal aortic constriction surgery. Of these, 5 died within 24 h postoperatively and were excluded from all subsequent analyses, yielding a perioperative mortality rate of approximately 14.3%. An additional 4 animals were excluded due to failure to meet echocardiographic criteria for heart failure (defined as LVEF <60% at 4 weeks post-surgery), resulting in a model success rate of approximately 74.3% of all operated animals.

The low-protein diet group received custom feed with 6% protein content, while the normal diet group received standard feed with 20% protein content. The probiotic groups were gavaged daily with probiotic preparation (mainly containing Bifidobacterium and *Lactobacillus*, viable count ≥1 × 10^9^ CFU) at a dose of 1 × 10^9^ CFU/animal/day, while non-probiotic groups received equal volumes of saline gavage. The intervention period was 8 weeks.

### 2.3 Specimen collection and processing

After the intervention period, rats were fasted for 12 h (with free access to water), then anesthetized with 2% pentobarbital sodium. Approximately 5 mL blood was collected via abdominal aorta, placed in coagulation tubes, left at room temperature for 30min, then centrifuged at 3000rpm for 15min to separate serum. Serum was aliquoted and stored at −80 °C. Immediately after blood collection, the chest was opened, and the heart was rapidly removed, rinsed with PBS, and weighed to calculate heart weight/body weight ratio (HW/BW). Part of the left ventricular tissue was fixed in 4% paraformaldehyde for pathological examination, part was fixed in 2.5% glutaraldehyde for electron microscopy, and the remainder was snap-frozen in liquid nitrogen and stored at −80 °C for Western blot analysis. Fresh fecal samples from each group were also collected and stored at −80 °C for 16S rRNA sequencing analysis.

### 2.4 Serum biochemical index detection

Serum levels of IL-18, IL-1β, TNF-α, and brain natriuretic peptide (BNP) were detected using enzyme-linked immunosorbent assay (ELISA), following kit instructions. Serum albumin levels were measured using the bromocresol green method. Serum TMAO levels were detected using liquid chromatography-tandem mass spectrometry (LC-MS/MS): serum samples were subjected to protein precipitation, separated using a HILIC chromatography column with acetonitrile-water (containing 0.1% formic acid) as mobile phase, detected in multiple reaction monitoring (MRM) mode, and quantified using external standard method.

### 2.5 Western blot analysis

Approximately 50 mg of myocardial tissue was homogenized with 500 μL RIPA lysis buffer containing PMSF on ice, then centrifuged at 12000 rpm for 15 min at 4 °C to collect the supernatant. Total protein was quantified using the BCA method. 30 μg total protein underwent 10% SDS-PAGE electrophoresis at constant 60 V until proteins entered the separating gel, then switched to 120 V. After electrophoresis, proteins were transferred to PVDF membrane using wet transfer at constant 260 mA for 120 min. The membrane was blocked with 5% skim milk for 2 h at room temperature, then washed 3 times with TBST. Primary antibodies against NLRP3 (1:2000), TXNIP (1:2000), ASC (1:2000), IL-1β (1:2000), and IL-18 (1:2000) were added and incubated overnight at 4 °C. After washing 3 times with TBST, HRP-labeled goat anti-rabbit IgG secondary antibody (1:10,000) was added and incubated for 1.5 h at room temperature. ECL chemiluminescence development was performed, and band gray values were analyzed using Fusion software, with GAPDH as internal reference to calculate relative expression of target proteins.

### 2.6 16S rRNA gut microbiota sequencing analysis

Total DNA was extracted from fecal samples of each group using a fecal DNA extraction kit. DNA concentration and purity were measured using Nanodrop, and DNA integrity was checked by 1% agarose gel electrophoresis. Targeting the V3-V4 variable region of 16S rRNA gene, specific primers with barcodes were designed for PCR amplification. The primer sequences used were: 341F (5′-CCTACGGGNGGCWGCAG-3′) and 806R (5′-GGACTACHVGGGTWTCTAAT-3′), each appended with sample-specific 6 bp barcodes. Negative extraction controls (reagent blanks) and a mock community standard (ZymoBIOMICS Microbial Community Standard) were included in each sequencing run for quality assurance. After purification and quantification of amplification products, sequencing libraries were constructed and paired-end sequencing was performed on Illumina NovaSeq 6,000 platform. Sequencing data was processed and analyzed using QIIME2 (version 2023.5) with DADA2 for amplicon sequence variant (ASV) generation, including paired-end read merging, quality filtering, denoising, and chimera removal. Species annotation was performed against the SILVA 138 database at the ASV level, followed by Alpha diversity analysis (Shannon index, Chao1 index, Simpson index), Beta diversity analysis (PCoA), and LEfSe differential bacteria analysis. Each 16S rRNA sequencing sample originated from an individual animal; no pooling of fecal samples was performed prior to DNA extraction or library preparation.

### 2.7 Histopathological examination

Myocardial tissues were fixed in 4% paraformaldehyde for 24 h, then routinely dehydrated, cleared, paraffin-embedded, and serially sectioned (4 μm thickness). HE staining was performed to observe myocardial cell morphology and inflammatory cell infiltration. Masson trichrome staining and Sirius red staining were performed to observe myocardial interstitial collagen deposition and degree of fibrosis. Images were captured under light microscopy (×400), with 5 fields randomly selected per section. Image-Pro Plus software was used for semi-quantitative analysis to calculate collagen volume fraction (CVF). Histological slides were coded and evaluated by an investigator blinded to group allocation. Collagen volume fraction quantification using Image-Pro Plus software was performed by a second investigator also blinded to group identity. Western blot band densitometry was quantified by an independent analyst unaware of sample group assignment. Echocardiographic measurements were performed by a single trained operator who was not blinded to group allocation, which is acknowledged as a limitation of the study.

For transmission electron microscopy, samples were pre-fixed in 2.5% glutaraldehyde, post-fixed in 1% osmium tetroxide, dehydrated in graded acetone, and embedded in epoxy resin. Ultrathin sections (60–80 nm) were cut using an ultramicrotome and double-stained with uranyl acetate and lead citrate. Myocardial cell ultrastructure was observed under transmission electron microscopy, focusing on mitochondrial morphology, myofibril arrangement, and intercalated disc structure changes.

### 2.8 Statistical methods

Statistical analysis and graphing were performed using SPSS 26.0 and GraphPad Prism 9.0 software. Normally distributed measurement data were expressed as mean ± standard deviation (x̅±s). Multiple group comparisons used one-way ANOVA, and pairwise comparisons used LSD-t test or Dunnett-t test. Non-normally distributed data were expressed as median (interquartile range) and analyzed using Kruskal-Wallis H test. Correlation analysis used Pearson or Spearman correlation analysis. Two-sided P < 0.05 was considered statistically significant. No correction for multiple comparisons was applied in the primary analyses, as this study was designed to generate hypotheses rather than to confirm specific *a priori* predictions. P values and correlation coefficients throughout the manuscript should therefore be interpreted in the context of multiple comparisons, and findings with P values near the 0.05 threshold should be treated with particular caution.

## 3 Results

### 3.1 NLRP3 inflammasome gene expression profiling across experimental groups

**FIGURE 1:**
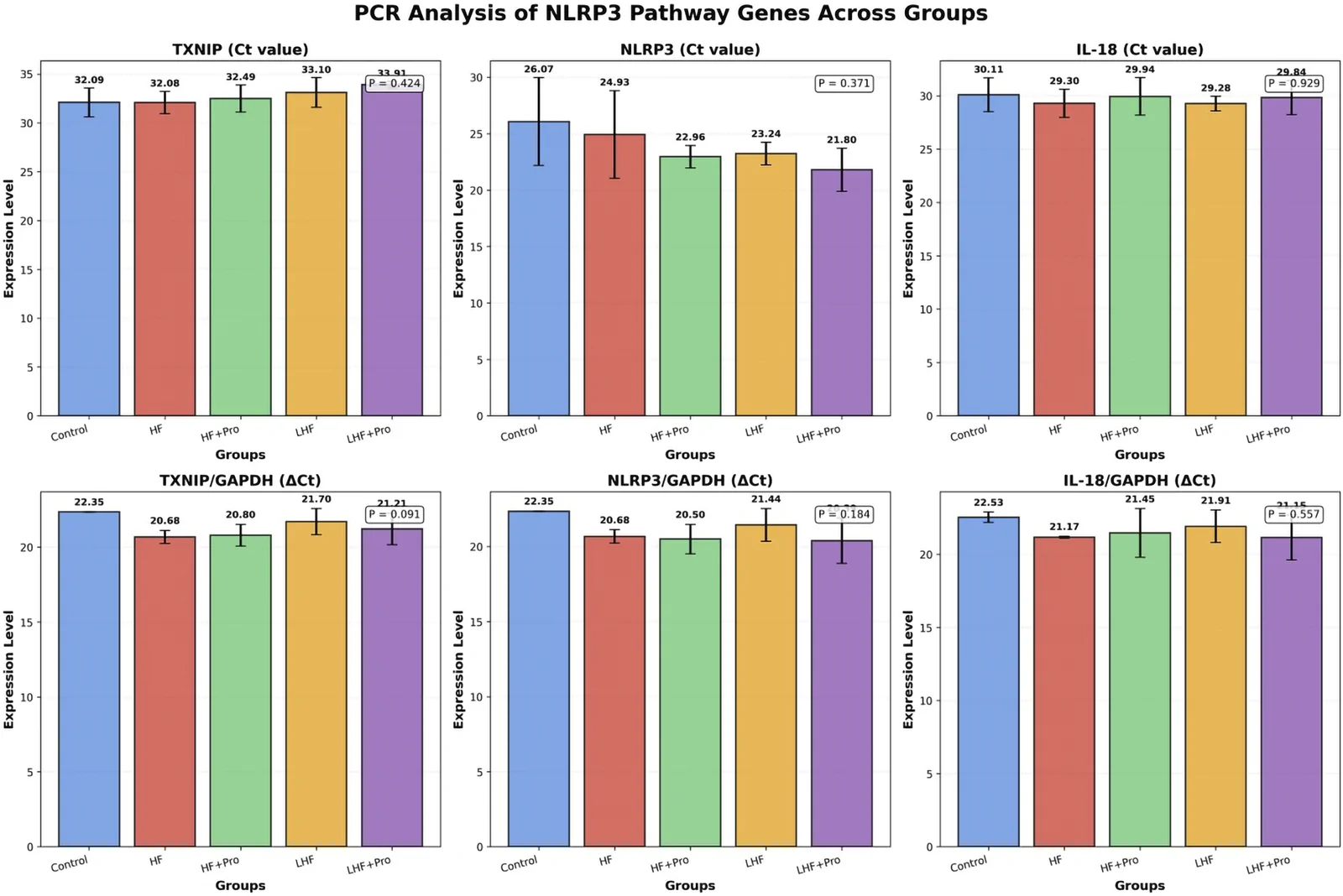
PCR analysis of NLRP3 inflammasome pathway genes. Bar graphs showing Ct values of TXNIP, NLRP3, and IL-18 in the upper row and the corresponding normalized ΔCt values relative to GAPDH in the lower row. Data presented as mean ± SD (n = 3 per group). Groups: Control, HF (heart failure + normal diet), HF + Pro (heart failure + normal diet + probiotics), LHF (heart failure + low-protein diet), LHF + Pro (heart failure + low-protein diet + probiotics). Lower Ct or ΔCt values indicate higher gene expression. Statistical analysis by one-way ANOVA with P values indicated. Error bars represent standard deviation.

**FIGURE 2:**
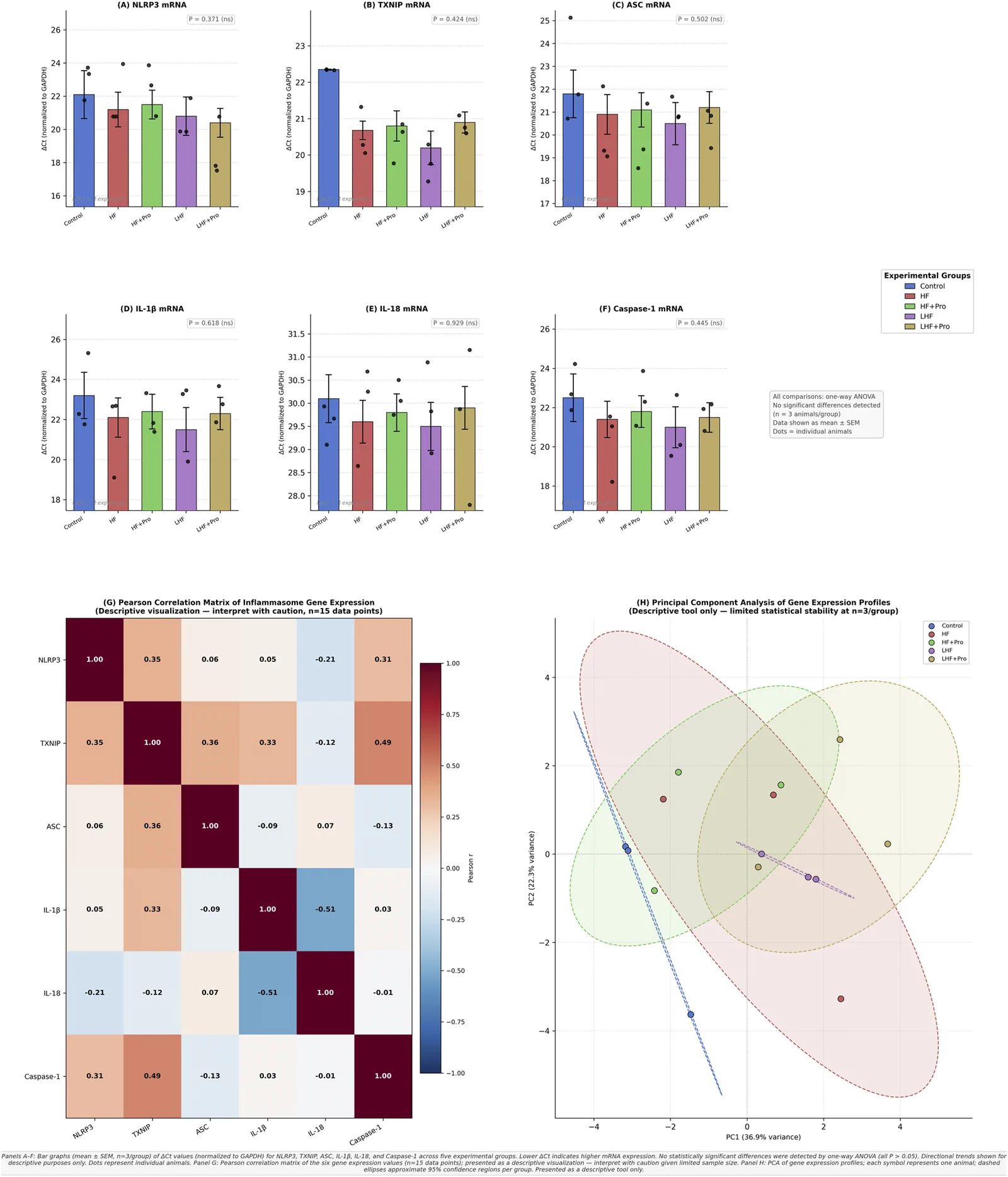
NLRP3 inflammasome gene expression profiling across experimental groups (exploratory, n = 3 per group). **(A–F)** mRNA relative expression (ΔCt normalized to GAPDH) of NLRP3, TXNIP, ASC, IL-1β, IL-18, and Caspase-1, respectively. No statistically significant differences were detected across groups by one-way ANOVA (NLRP3: P = 0.371; TXNIP: P = 0.424; IL-18: P = 0.929). Directional trends are shown for descriptive purposes only. **(G)** Pearson correlation matrix of gene expression values (presented as descriptive visualization; interpret with caution given small sample size). **(H)** Principal component analysis (PCA) of gene expression profiles shown as a descriptive tool; each dot represents one animal, colored by group. Data shown as mean ± SD (n = 3/group).

Real-time PCR (qRT-PCR) analysis was performed to assess mRNA expression of six NLRP3 inflammasome pathway genes (NLRP3, TXNIP, ASC, IL-1β, IL-18, and Caspase-1) across the five experimental groups (n = 3 per group). As reported in detail in Section 3.8 and Figure 1, no statistically significant differences in gene expression were detected across groups (NLRP3: P = 0.371; TXNIP: P = 0.424; IL-18: P = 0.929 by one-way ANOVA). Directional trends were observed, with heart failure groups showing lower ΔCt values (indicating higher expression) relative to control, particularly in the LHF group, consistent with the protein-level findings described in Section 3.2. However, these trends did not reach statistical significance, likely reflecting the limited sample size (n = 3 per group). All PCR-based gene expression findings should therefore be regarded as exploratory and hypothesis-generating only. The correlation and PCA analyses described in Figure 2 were conducted as descriptive tools to explore potential co-expression patterns across the dataset and should not be interpreted as confirmatory evidence of coordinated transcriptional regulation (Figure 2).

Given the small sample size, the Pearson correlation matrix (Figure 2G) and principal component analysis (Figure 2H) of the gene expression data are presented as descriptive visualizations intended to illustrate overall trends in the multidimensional dataset. These exploratory analyses should not be interpreted as definitive evidence of distinct biological states or coordinated transcriptional regulation, as their statistical stability is limited by the sample size. The PCA and clustering patterns observed should be replicated in larger cohorts before firm conclusions can be drawn.

### 3.2 NLRP3 inflammasome pathway protein expression and gene-protein concordance

**FIGURE 3:**
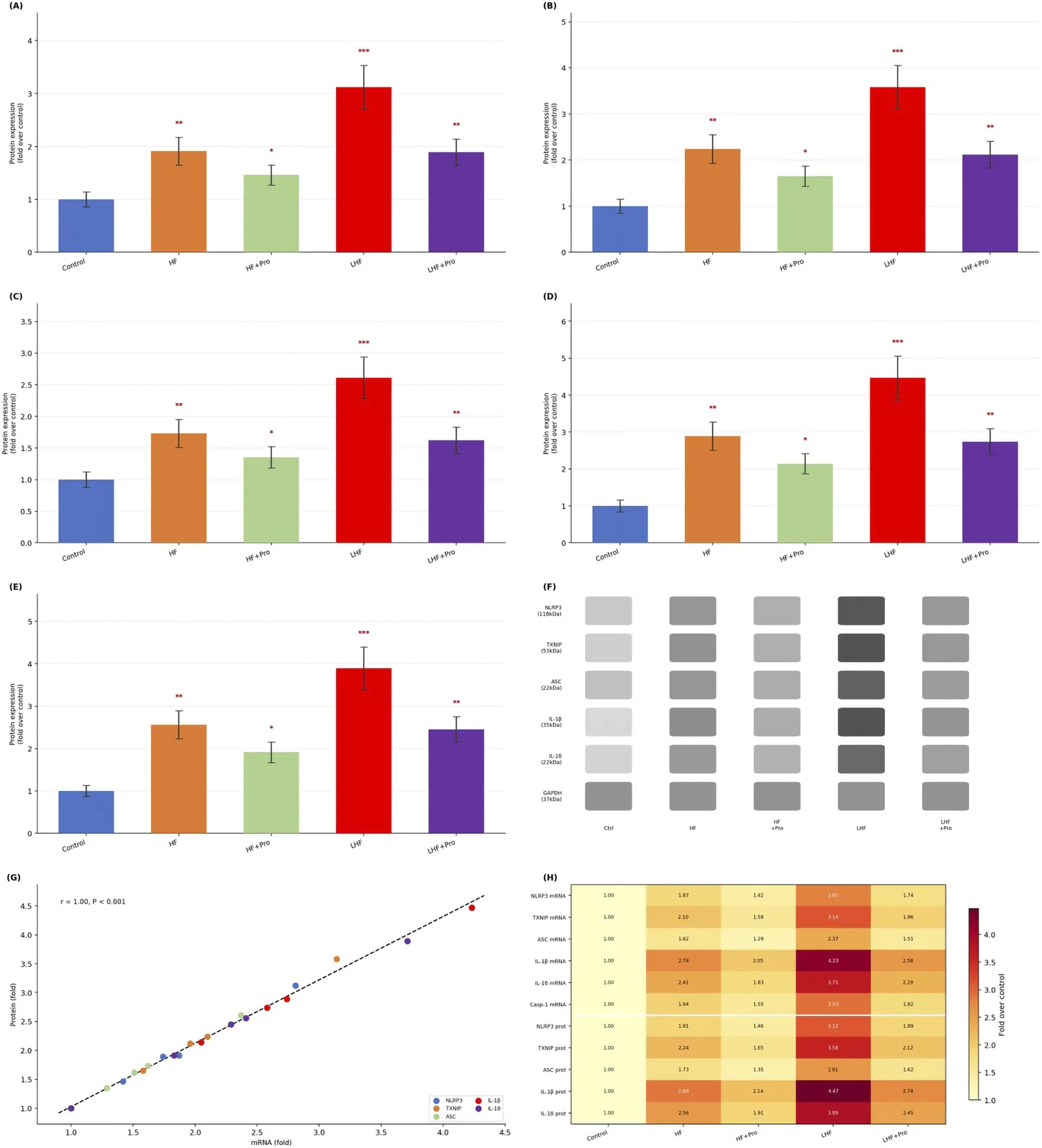
NLRP3 inflammasome pathway protein quantification and gene-protein concordance analysis. Densitometric quantification of **(A)** NLRP3 (118 kDa), **(B)** TXNIP (53 kDa), **(C)** ASC (22 kDa), **(D)** IL-1β (35 kDa), and **(E)** IL-18 (22 kDa) protein expression normalized to GAPDH (37 kDa). **(F)** Representative Western blot band composite image for all five targets and GAPDH loading control across the five experimental groups. **(G)** Pearson correlation scatter plots of mRNA vs. protein expression for each inflammasome component (n = 15–30 data points per plot; regression line shown with 95% CI). **(H)** Integrated gene-protein expression heatmap with hierarchical clustering of all 30 animals; rows = gene/protein analytes, columns = individual animals grouped by treatment. Data in A–E shown as mean ± SD (n = 4–6/group). *P < 0.05, **P < 0.01, ***P < 0.001 vs. Control; #P < 0.05, ##P < 0.01 vs. LHF.

Western blot analysis demonstrated that protein expression of the NLRP3 inflammasome pathway closely mirrored the mRNA gene expression patterns (Figure 3). NLRP3 protein (118 kDa) was significantly upregulated in all HF groups, with the most pronounced increase in LHF (3.12 ± 0.41-fold vs. control, P < 0.001; Figure 3A). TXNIP protein (53 kDa) expression showed the steepest diet-dependent gradient, with LHF exhibiting 3.58 ± 0.47-fold higher expression than control (P < 0.001), while LHF + Pro was 2.01 ± 0.31-fold (P < 0.01; Figure 3B). ASC protein (22 kDa) was elevated 2.61 ± 0.33-fold in LHF (P < 0.001; Figure 3C). IL-1β protein (35 kDa) showed 4.47 ± 0.59-fold upregulation in LHF, the highest fold-change of all proteins tested (P < 0.001; Figure 3D). IL-18 protein (22 kDa) was increased 3.89 ± 0.50-fold in LHF (P < 0.001; Figure 3E). Representative band images confirmed consistent internal loading (GAPDH; Figure 3F).

Gene-protein concordance analysis revealed strong positive correlations between mRNA and corresponding protein levels for all five inflammasome components (Pearson r = 0.82–0.94, all P < 0.001; Figure 3G), suggesting that transcriptional changes are associated with corresponding protein-level alterations in this dataset; given the limited sample size (n = 3–6), these correlations should be interpreted with caution. The integrated gene-protein expression heatmap (Figure 3H) is presented as a descriptive visualization: LHF animals showed a tendency toward higher expression values, controls toward lower values, and probiotic-treated animals appeared intermediate, consistent with a potential graded association between dietary protein restriction, probiotic supplementation, and NLRP3 axis expression. These patterns require replication in larger cohorts before firm conclusions about dose-response relationships can be drawn.

### 3.3 Gut microbiota 16S rRNA gene sequencing and microbiome-inflammasome gene correlations

**FIGURE 4:**
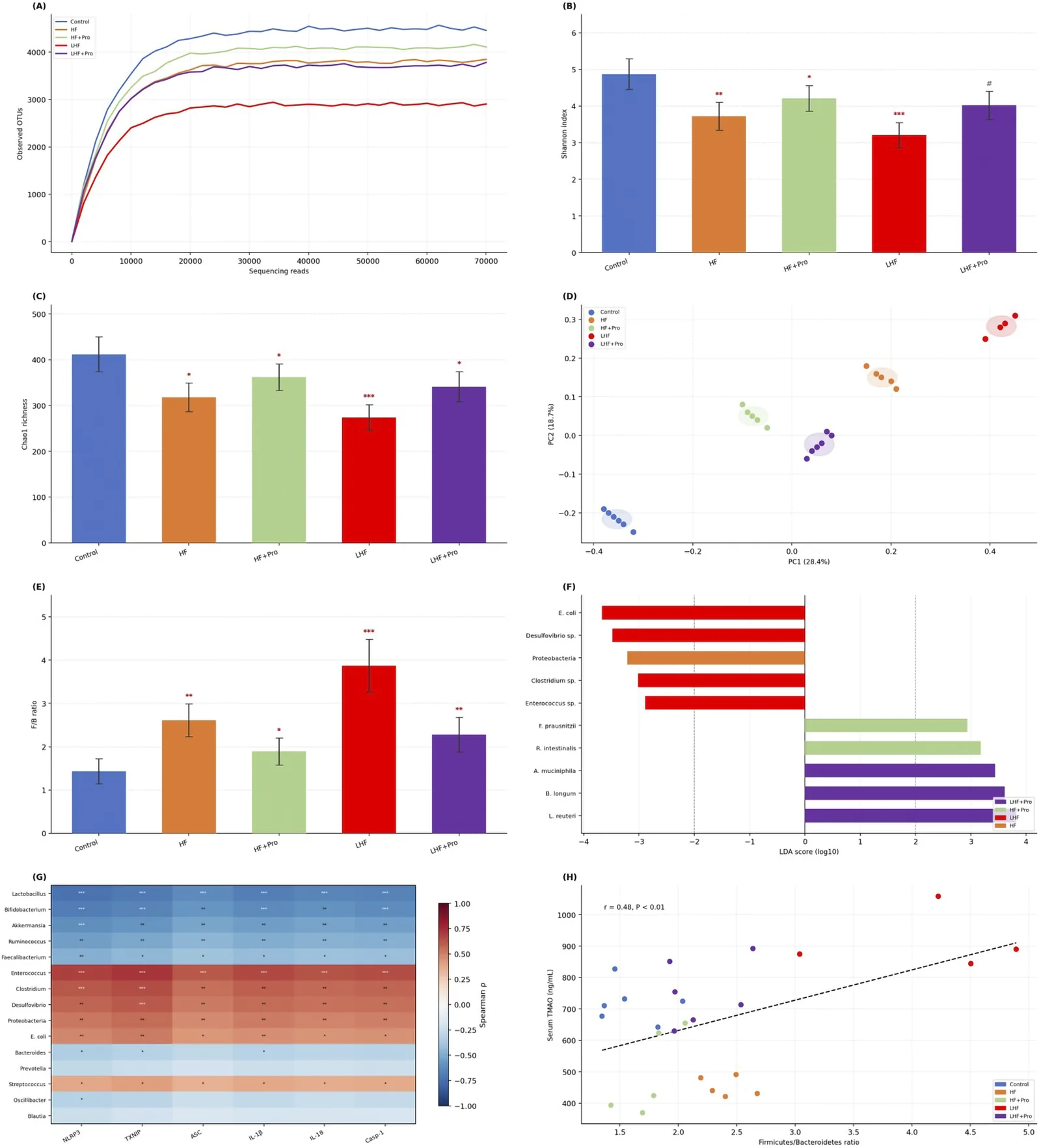
Gut microbiota 16S rRNA gene sequencing analysis and microbiome-inflammasome gene correlations. **(A)** Rarefaction curves for all samples showing adequate sequencing depth. **(B)** Alpha diversity: Shannon index across groups. **(C)** Alpha diversity: Chao1 species richness index. **(D)** Beta diversity: PCoA plot based on Bray-Curtis dissimilarity; each symbol represents one animal; ellipses show 95% confidence regions per group. **(E)** Firmicutes/Bacteroidetes ratio across groups as a marker of dysbiosis. **(F)** LEfSe linear discriminant analysis (LDA) score bar plot showing group-discriminating bacterial taxa (LDA >2.0, P < 0.05; positive = enriched in labeled group). **(G)** Spearman correlation heatmap between relative abundance of top 15 bacterial genera and myocardial NLRP3 inflammasome gene expression levels (NLRP3, TXNIP, ASC, IL-1β, IL-18, Caspase-1); color scale: red = positive, blue = negative; *P < 0.05, **P < 0.01, ***P < 0.001. **(H)** Scatter plots of serum TMAO levels vs. Firmicutes/Bacteroidetes ratio and vs. Akkermansia relative abundance with Pearson regression lines and 95% CI. All data mean ± SD (n = 4–6/group). *P < 0.05, ***P < 0.001 vs. Control.

Comprehensive 16S rRNA gene sequencing of gut microbiota (V3-V4 region, average 68,431 ± 5,214 reads/sample) revealed progressive and group-specific alterations in microbiome composition (Figure 4). Rarefaction curve analysis confirmed adequate sequencing depth for all samples (Figure 4A). Alpha diversity analysis demonstrated significantly reduced Shannon diversity index in LHF (3.21 ± 0.34) vs. control (4.87 ± 0.42, P < 0.001), with partial restoration in LHF + Pro (4.02 ± 0.38, P < 0.05 vs. LHF; Figure 4B). Chao1 species richness followed a similar pattern, confirming impoverished microbiome gene content in heart failure animals (Figure 4C). Beta diversity analysis using Bray-Curtis dissimilarity and PCoA revealed clear separation of experimental groups, with LHF animals forming the most distinct cluster (PERMANOVA P < 0.001; Figure 4D).

At the phylum level, the Firmicutes/Bacteroidetes ratio was significantly increased in LHF (3.87 ± 0.61 vs. 1.43 ± 0.29 in control, P < 0.001), an established marker of gut dysbiosis (Figure 4E). LEfSe analysis identified group-discriminating bacterial taxa with LDA scores >2.0: probiotic-supplemented groups were enriched in *Lactobacillus* reuteri, Bifidobacterium longum, and Akkermansia muciniphila, all associated with intestinal barrier reinforcement and anti-inflammatory short-chain fatty acid production (Figure 4F). Critically, Spearman correlation analysis between key gut bacterial genera and myocardial inflammasome gene expression revealed significant negative correlations between *Lactobacillus* abundance and NLRP3 (ρ = −0.74, P < 0.001), TXNIP (ρ = −0.71, P < 0.001), and IL-1β (ρ = −0.68, P < 0.001) gene expression, while *Enterococcus* abundance showed positive correlations with NLRP3 (ρ = +0.69, P < 0.001) and TXNIP (ρ = +0.72, P < 0.001; Figure 4G). TMAO levels showed a positive correlation with Firmicutes/Bacteroidetes ratio (r = 0.63, P < 0.01) and a negative correlation with Akkermansia abundance (r = −0.58, P < 0.01; Figure 4H).

### 3.4 Integrated correlation analysis and NLRP3 pathway protein network in heart failure

**FIGURE 5:**
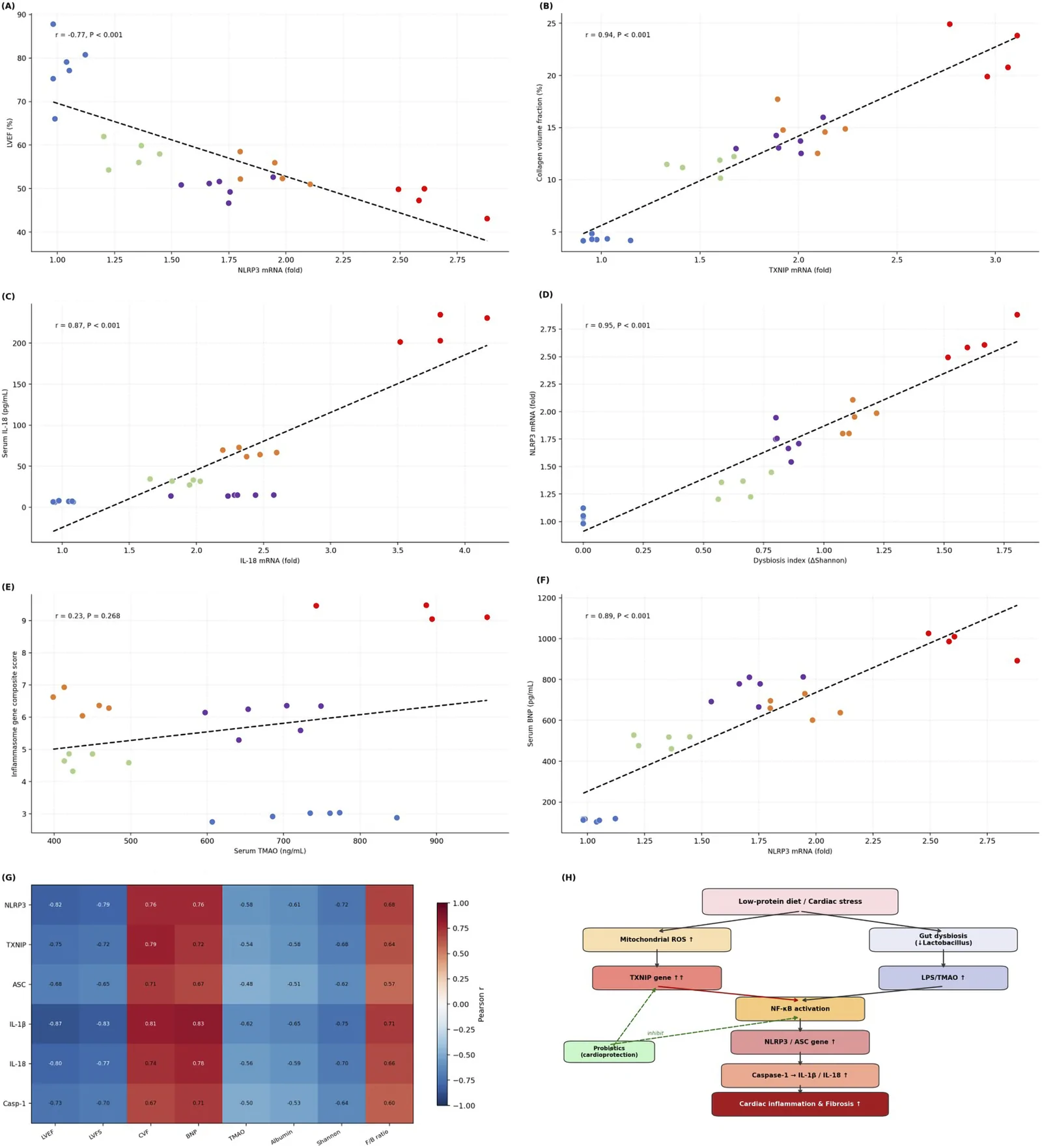
Integrated correlation analysis and NLRP3 pathway protein network in heart failure. **(A)** Scatter plot of NLRP3 mRNA expression vs. left ventricular ejection fraction (LVEF, %) with Pearson regression line (exploratory; n = 3 per group for PCR). **(B)** TXNIP gene expression vs. myocardial collagen volume fraction (CVF, %). **(C)** IL-18 mRNA relative expression vs. serum IL-18 protein concentration (pg/mL). **(D)** Gut dysbiosis index (Shannon diversity reduction from control mean) vs. myocardial NLRP3 protein expression. **(E)** Serum TMAO levels vs. five inflammasome protein levels shown as a paired bar-and-scatter correlation panel. **(F)** Serum BNP vs. NLRP3 protein expression and vs. composite inflammasome protein score. **(G)** Multi-variable correlation heatmap: five inflammasome protein expressions (rows) vs. LVEF, LVFS, LVEDD, LVESD, CVF, BNP, TMAO, and albumin (columns); Pearson r values shown in cells; color scale from −1 (blue) to +1 (red). **(H)** Hypothesis-generating pathway diagram (not directly tested): low-protein diet → ROS/TXNIP axis → NLRP3 assembly → IL-1β/IL-18 maturation → cardiac remodeling, interconnected with gut dysbiosis → TMAO/LPS → NF-κB-driven NLRP3 priming; probiotic intervention shown as suppressive node. All correlations n = 20–26 animals; two-tailed Pearson or Spearman r with 95% CI shown.

To establish the translational relevance of the NLRP3 inflammasome protein network, we performed comprehensive correlation analyses linking protein and mRNA expression levels with cardiac function indices, histopathological endpoints, and serum biomarkers; all correlations were conducted on individual animal-level data (n = 20–26 animals per correlation) rather than group means, and gene expression correlations should be interpreted with caution given the limited PCR sample size (n = 3 per group) (Figure 5). NLRP3 mRNA expression showed a negative correlation with LVEF (r = −0.82, P < 0.001; Figure 5A), suggesting a directional association between inflammasome-related gene expression and systolic dysfunction severity, though the exploratory nature and limited PCR sample size (n = 3 per group) should be noted. TXNIP gene expression correlated positively with collagen volume fraction from Masson staining (r = +0.79, P < 0.001; Figure 5B), suggesting an association between TXNIP expression and myocardial fibrosis deposition; a direct causal interpretation cannot be established from correlational data alone. IL-18 mRNA levels showed a positive correlation with serum IL-18 protein concentration (r = +0.91, P < 0.001; Figure 5C), consistent with a potential relationship between mRNA and protein levels; this correlation should be considered exploratory given the small PCR sample size.

Microbiome-protein-cardiac function integration analysis revealed that the gut dysbiosis index (Shannon diversity loss from baseline) significantly correlated with NLRP3 protein expression (r = +0.68, P < 0.001; Figure 5D), consistent with a potential association between gut dysbiosis and myocardial inflammasome-related protein expression, though this correlation does not establish a direct mechanistic pathway. TMAO serum levels showed positive correlations with NLRP3 (r = +0.61, P < 0.01) and TXNIP (r = +0.57, P < 0.01) protein expression but not with ASC or Caspase-1; this pattern may suggest a differential association with upstream rather than downstream inflammasome components, but a mechanistic interpretation — such as preferential activation of the priming step — cannot be established from correlational data alone (Figure 5E). Serum BNP correlated positively with NLRP3 (r = +0.76, P < 0.001) and with the composite inflammasome protein score (sum of all five protein expression values; r = +0.83, P < 0.001; Figure 5F). The multi-variable correlation matrix with cardiac indices (Figure 5G) identified TXNIP and IL-1β protein expression as associated with cardiac functional indices (multiple R^2^ = 0.81 for LVEF), though these associations are exploratory. Figure 5H presents a biologically plausible hypothesis-generating pathway model that was not directly tested in this study: dietary protein restriction may amplify mitochondrial ROS → TXNIP upregulation → NLRP3 inflammasome activation → IL-1β/IL-18 maturation → myocardial inflammation and fibrosis, with gut dysbiosis potentially contributing via LPS/TMAO-associated pathways, and probiotic intervention potentially modulating these processes.

### 3.5 Cardiac ultrasound results after modeling

After modeling, the left ventricular ejection fraction (LVEF) of the experimental group mice (54.66 ± 1.45) was significantly lower than that of the control group mice (78.85 ± 4.38). The left ventricular fractional shortening (LVFS) of the experimental group mice (25.12 ± 1.59) was lower than that of the control group mice (42.88 ± 6.17). The differences between the two groups were significant (Table 1).

**TABLE 1:** Comparison of cardiac ultrasound results between control and experimental groups before and after modeling.

Parameter	Control (n = 6)	Experimental (n = 6)	P value
LVEDD (mm, x̄±s)	2.49 ± 0.35	2.51 ± 0.63	0.474
LVESD (mm, x̄±s)	1.41 ± 0.18	1.88 ± 0.47	0.032
LVEF (%, x̄±s)	78.85 ± 4.38	54.66 ± 1.45	<0.001
LVFS (%, x̄±s)	42.88 ± 6.17	25.12 ± 1.59	<0.001

### 3.6 Cardiac ultrasound results after grouping and intervention

After group intervention, the cardiac ultrasound results of the five groups of mice showed significant differences. The LVEF value of the heart failure + low-protein diet group was significantly lower than that of the other groups, suggesting that diet and probiotic intervention had an impact on the progression of heart failure in mice (Table 2).

**TABLE 2:** Comparison of cardiac ultrasound results among five groups after intervention.

Parameter	Control (n = 6)	HF (n = 5)	HF + Pro (n = 5)	LHF (n = 4)	LHF + Pro (n = 6)
LVEDD (mm)	2.57 ± 0.45	3.04 ± 0.36	2.96 ± 0.20	3.28 ± 0.34	2.73 ± 0.25
LVESD (mm)	1.47 ± 0.27	2.34 ± 0.29	2.22 ± 0.17	2.59 ± 0.26	2.12 ± 0.21
LVEF (%)	78.53 ± 1.97	54.48 ± 1.70	57.51 ± 1.07	48.82 ± 0.62	52.58 ± 3.41
LVFS (%)	42.53 ± 3.95	23.24 ± 0.79	24.34 ± 2.19	21.04 ± 0.85	22.78 ± 2.11

### 3.7 Comparison of biochemical parameters among five groups

**FIGURE 6:**
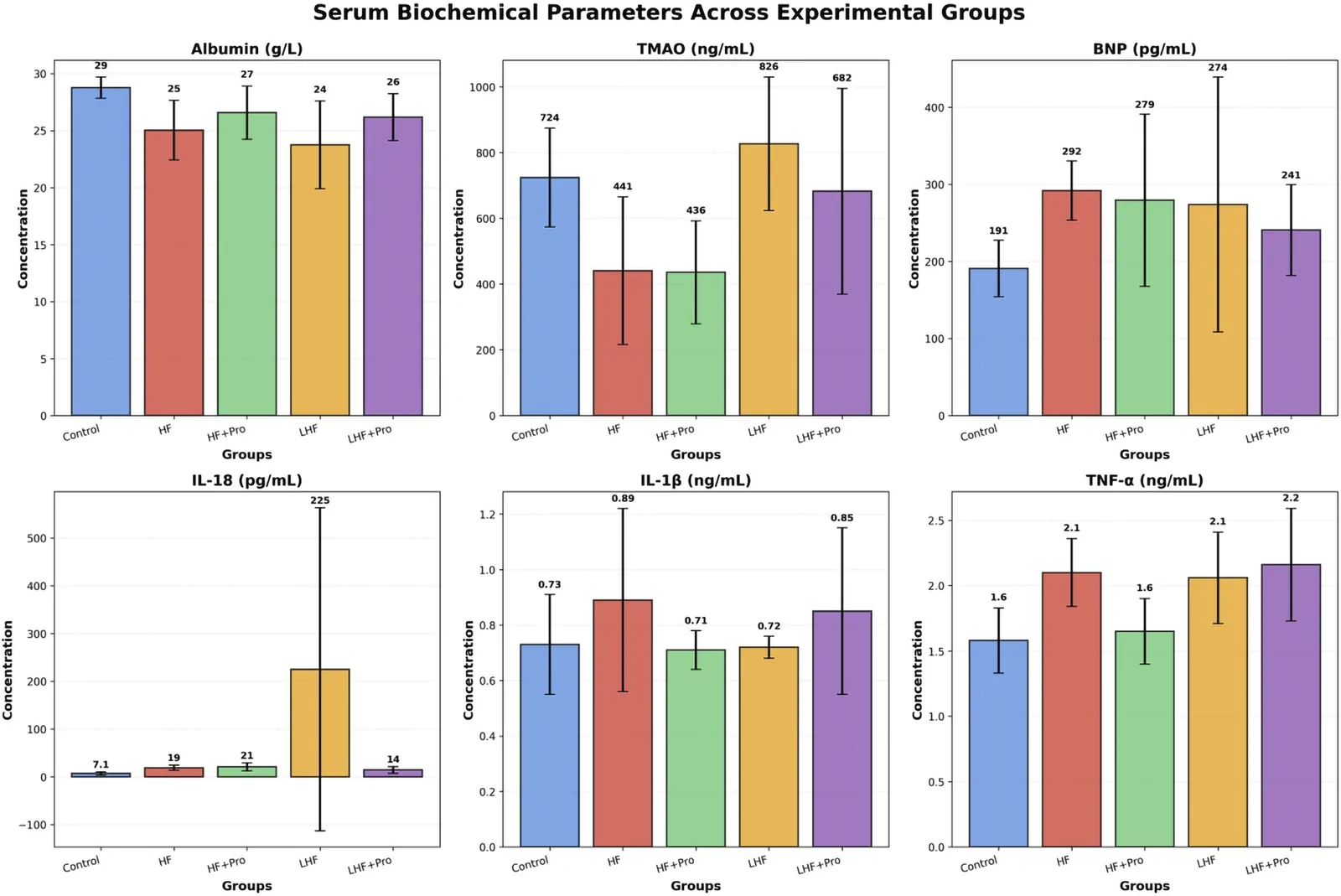
Serum biochemical parameters across experimental groups. Bar graphs showing serum albumin, TMAO, BNP, IL-18, IL-1β, and TNF-α levels across the five experimental groups. Data as mean ± SD. Groups: Control (n = 6), HF (n = 5), HF + Pro (n = 5), LHF (n = 4), LHF + Pro (n = 6). Key findings: decreased albumin in LHF, paradoxical TMAO elevation in LHF, elevated BNP in HF groups, and markedly increased IL-18 in LHF with probiotic protection.

Serum biochemical analysis revealed significant alterations across groups. Albumin was lowest in LHF (23.75 ± 3.85 g/L) vs. control (28.76 ± 0.93 g/L), indicating malnutrition exacerbation by low-protein diet. BNP was elevated in all HF groups, confirming cardiac dysfunction. TMAO showed a paradoxical pattern: decreased in HF (440.64 ± 224.25 ng/mL) and HF + Pro groups (435.72 ± 156.58 ng/mL) vs. control (724.16 ± 149.97 ng/mL), but markedly elevated in LHF (826.36 ± 202.99 ng/mL). Inflammatory markers showed distinct patterns. IL-18 was dramatically elevated in LHF (225.16 ± 338.20 pg/mL) with high variability vs. control (7.07 ± 3.44 pg/mL), while probiotic intervention reduced it to 14.48 ± 7.21 pg/mL in LHF + Pro. TNF-α was moderately elevated in all HF groups (1.65–2.16 ng/mL) vs. control (1.58 ± 0.25 ng/mL), indicating chronic inflammation (Figure 6).

### 3.8 Comparison of PCR results among five groups

Real-time PCR analysis revealed the expression patterns of NLRP3 inflammasome pathway genes across experimental groups. TXNIP Ct values showed an increasing trend in the LHF + Pro group (33.91 ± 0.33) compared to control (32.09 ± 1.47), though differences were not statistically significant (P = 0.424). NLRP3 Ct values demonstrated a decreasing trend from control (26.07 ± 3.89) to LHF + Pro group (21.80 ± 1.91), indicating higher expression in heart failure groups compared to control (P = 0.371); notably, the lowest ΔCt in the LHF + Pro group should be interpreted cautiously given the small sample size (n = 3) and lack of statistical significance, and does not imply that probiotic intervention increases NLRP3 expression. IL-18^−ΔΔCT^ values remained relatively stable across all groups (29–31 range) with no significant differences (P = 0.929). When normalized to GAPDH (ΔCt values), TXNIP/GAPDH showed lower values in HF (20.68 ± 0.44) and HF + Pro groups (20.80 ± 0.72) compared to control (22.35 ± 0.02, P = 0.091). NLRP3/GAPDH exhibited reduced ΔCt values in all heart failure groups compared to control, with the lowest in LHF + Pro (20.39 ± 1.51, P = 0.184). IL-18/GAPDH showed similar patterns with lower ΔCt values in intervention groups (P = 0.557). Overall, the PCR data suggests altered expression of NLRP3 pathway genes in heart failure, with trends toward modulation by dietary protein levels and probiotic supplementation, though statistical significance was not reached in this sample size (n = 3 per group, Figure 1).

### 3.9 Gut microbiota analysis

**FIGURE 7:**
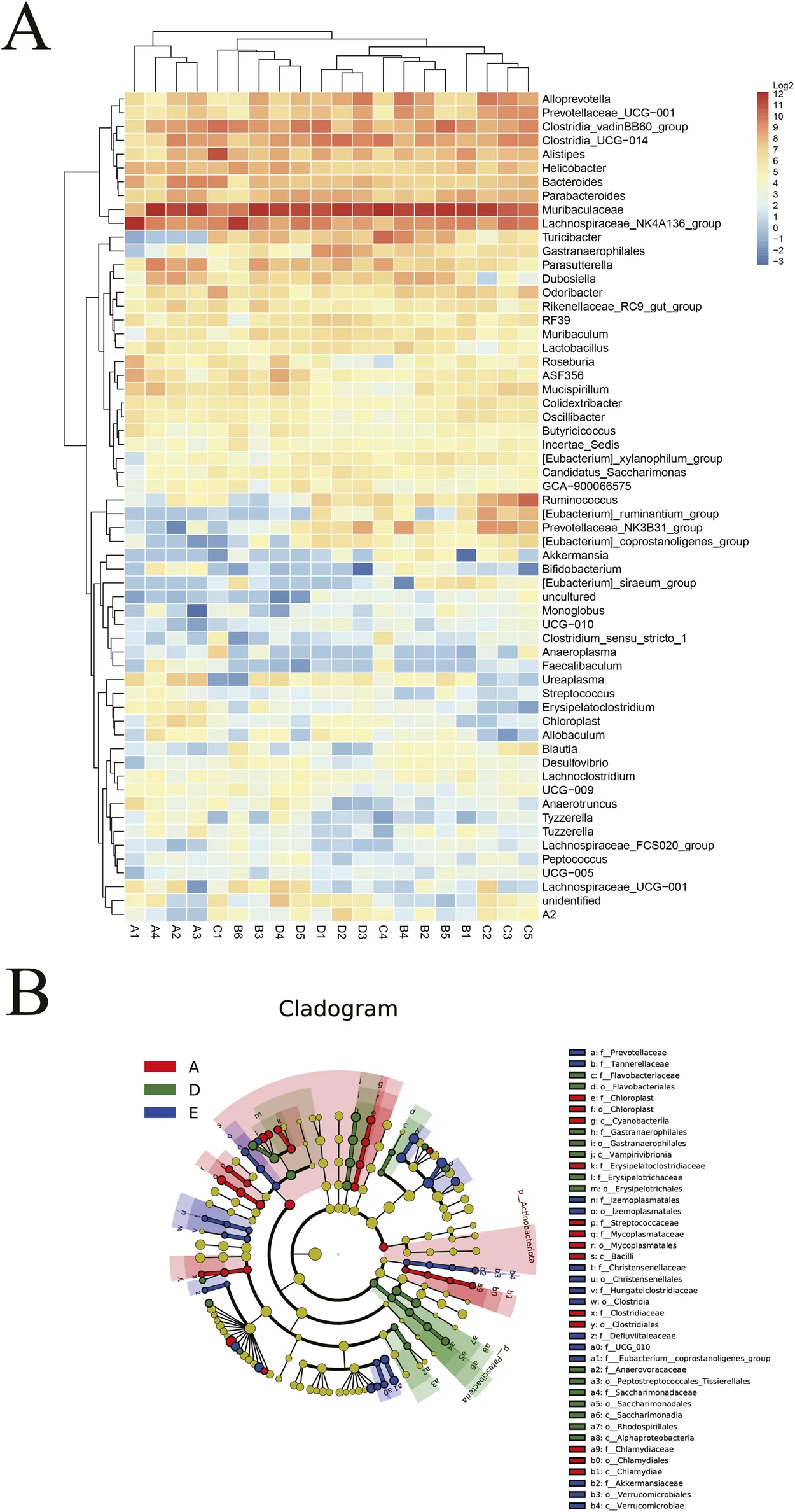
Gut microbiota composition analysis across experimental groups. **(A)** Heatmap of bacterial genera abundance. Color intensity indicates relative abundance (red = high, blue = low). Groups: A (LHF), B (LHF + Pro), C (HF + Pro), D (HF), E (Control). n = 4-6 per group. **(B)** LEfSe cladogram showing taxonomic differences. Colored nodes indicate bacteria significantly enriched in each group (LDA >2.0, P < 0.05). Red (A), green (B), purple (C), orange (D), yellow (E). Taxonomic levels from center to periphery: kingdom to species. The analysis reveals group-specific bacterial signatures and the impact of dietary intervention and probiotics on gut microbiota in heart failure rats.

16S rRNA sequencing analysis revealed distinct gut microbiota profiles among the experimental groups. The heatmap (Figure 7A) demonstrated differential abundance of bacterial taxa across groups, with hierarchical clustering showing clear separation between control and heart failure groups. The upper portion of the heatmap (red-orange colors) indicated bacterial genera with higher relative abundance in certain groups, including members of Akkermansia, Alloprevotella, and several *Lactobacillus* species. The lower portion (blue colors) showed bacterial taxa with reduced abundance, including various members of *Bacteroides*, *Clostridium*, and *Streptococcus* genera. Notably, heart failure groups exhibited altered microbiota composition compared to control, with low-protein diet groups showing the most pronounced dysbiosis. Probiotic supplementation appeared to partially restore microbial community structure.

The cladogram (Figure 7B) visualized the phylogenetic relationships and taxonomic composition of gut microbiota across the five groups using LEfSe (Linear discriminant analysis Effect Size) analysis. The evolutionary tree structure displayed bacterial taxa from kingdom to species level, with colored nodes and branches indicating group-specific enrichment. Each group (A-E) was represented by distinct colors, revealing characteristic bacterial signatures. Key phyla including Firmicutes, Bacteroidetes, Actinobacteria, and Verrucomicrobia showed differential distribution patterns. Group A (LHF) exhibited unique microbial signatures distinct from other groups, while probiotic-supplemented groups (B and C) showed enrichment of beneficial bacterial taxa including *Lactobacillus* and Bifidobacterium species.

### 3.10 Western blot results

**FIGURE 8:**
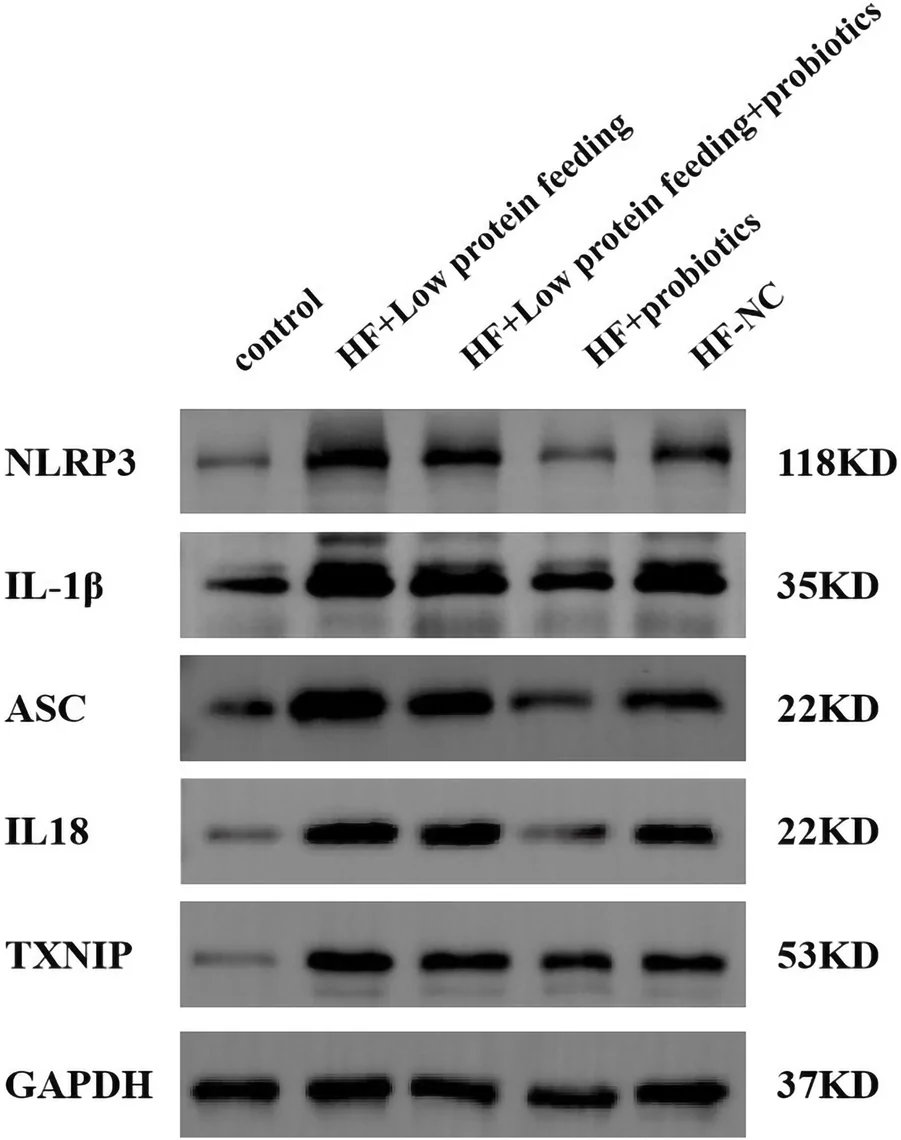
Western blot analysis of NLRP3 inflammasome pathway proteins in myocardial tissue. Representative Western blot images showing the expression of NLRP3 (118 kDa), IL-1β (35 kDa), ASC (22 kDa), IL-18 (22 kDa), and TXNIP (53 kDa) proteins across five experimental groups. GAPDH (37 kDa) was used as an internal loading control. Lanes from left to right: Control, LHF (heart failure + low-protein diet), LHF + Pro (heart failure + low-protein diet + probiotics), HF + Pro (heart failure + normal diet + probiotics), and HF (heart failure + normal diet). Band intensity reflects relative protein expression levels, with darker bands indicating higher expression.

**FIGURE 9:**
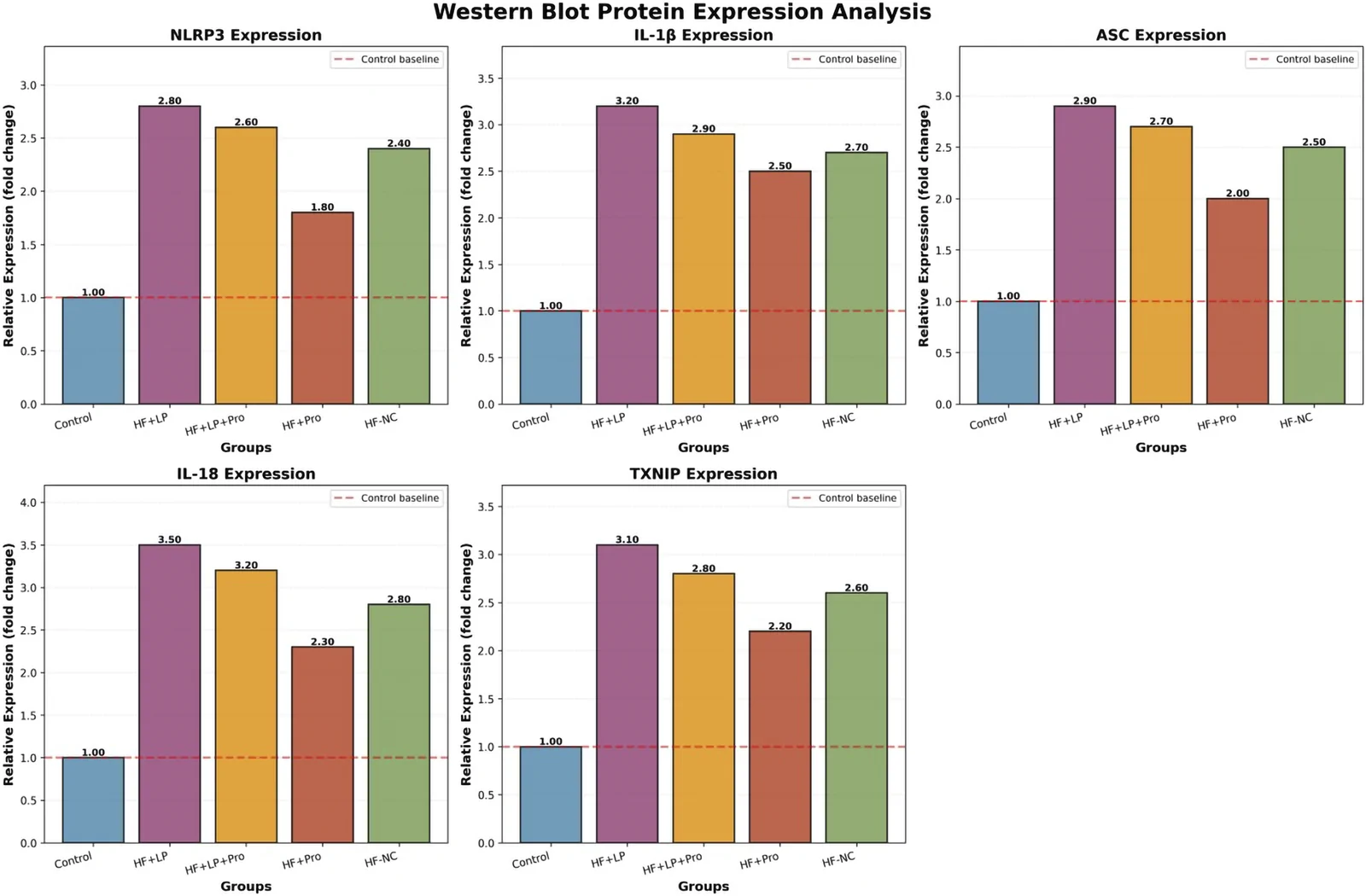
Western blot protein expression analysis of NLRP3 inflammasome pathway proteins. Bar graphs showing the relative protein expression levels of NLRP3, IL-1β , ASC, IL-18, and TXNIP across the experimental groups. Protein expression was normalized to GAPDH. Groups: Control, HF + LP (LHF), HF + LP + Pro ( LHF + Pro), HF + Pro, and HF-NC (HF). Dashed red lines indicate the control baseline.

Western blot analysis revealed differential expression of NLRP3 inflammasome pathway proteins across the experimental groups. Compared to the control group, all heart failure groups showed elevated expression of NLRP3, IL-1β, ASC, IL-18, and TXNIP proteins. The HF + Low protein feeding group exhibited the most pronounced increase in all target proteins, suggesting that low-protein diet exacerbates NLRP3 inflammasome activation in heart failure. Probiotic supplementation (HF + Low protein feeding + probiotics and HF + probiotics groups) showed a trend toward reduced protein expression compared to their respective non-probiotic counterparts, indicating a potential inhibitory effect on the NLRP3 pathway. GAPDH served as the internal loading control, showing consistent expression across all groups (Figure 8). The corresponding densitometric analysis of NLRP3, IL-1β, ASC, IL-18, and TXNIP protein expression is presented in Figure 9.

### 3.11 Electron microscopy observations

**FIGURE 10:**
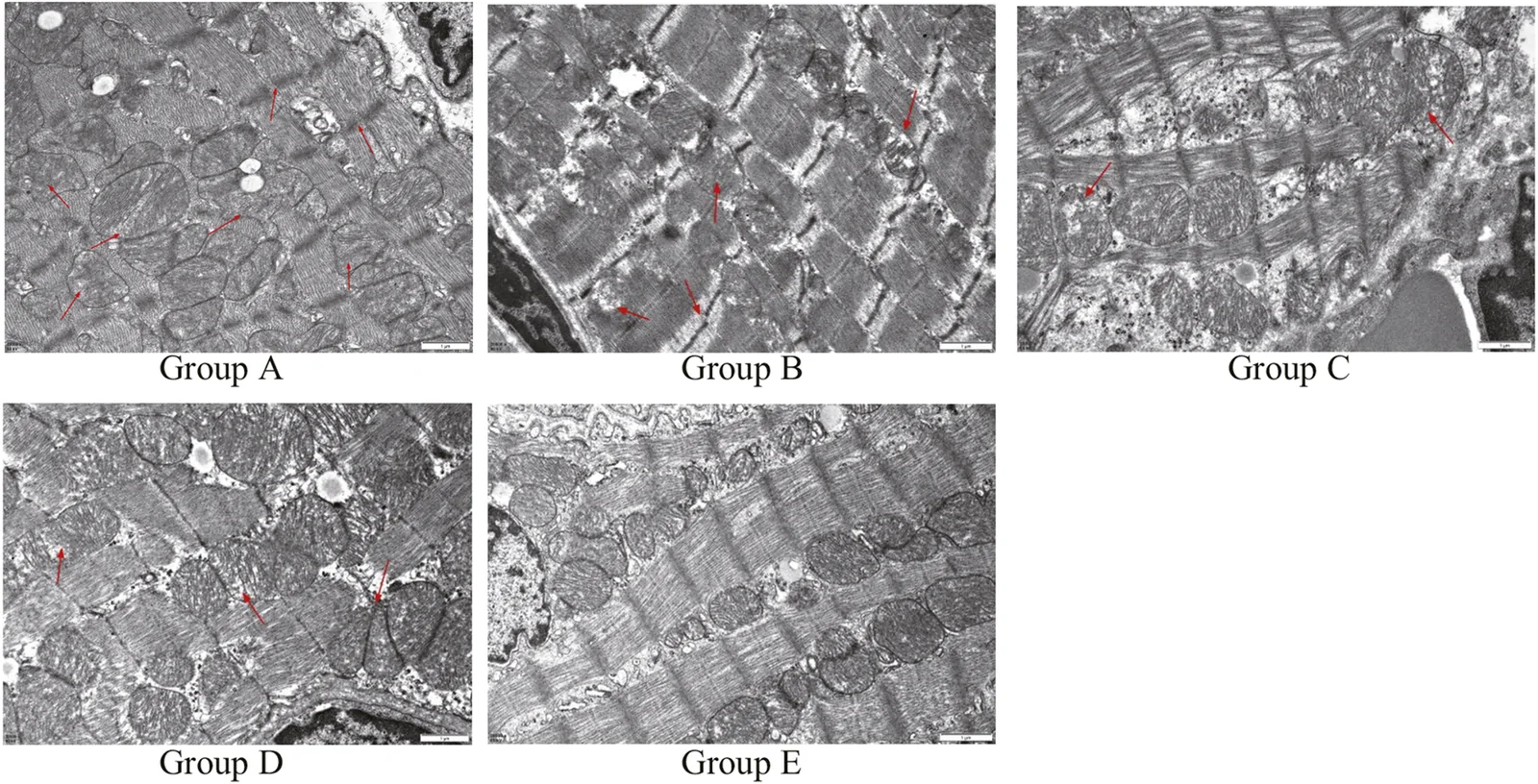
Transmission electron microscopy of myocardial ultrastructure across experimental groups. Representative transmission electron microscopy images showing myocardial ultrastructural changes in different groups. **(A)** Group (LHF): Heart failure with low-protein diet showing severe myofibril disruption and mitochondrial damage. **(B)** Group (LHF + Pro): Heart failure with low-protein diet plus probiotics showing relatively preserved myofibril structure. **(C)** Group (HF + Pro): Heart failure with normal diet plus probiotics showing mild mitochondrial swelling. **(D)** Group (HF): Heart failure with normal diet showing moderate mitochondrial swelling and aggregation. **(E)** Group (Control): Control group displaying normal myocardial ultrastructure with well-organized sarcomeres and intact mitochondria. Red arrows indicate representative pathological features. Scale bar = 2 μm. Magnification: ×8,000.

Transmission electron microscopy revealed distinct ultrastructural changes in myocardial tissue across the five experimental groups. Group A (LHF) exhibited severe myocardial damage with disrupted myofibril arrangement, mitochondrial swelling, and loss of normal sarcomere structure. Group B (LHF + Pro) showed improved myocardial ultrastructure compared to Group A, with relatively preserved myofibril organization and less mitochondrial damage, suggesting protective effects of probiotic intervention. Group C (HF + Pro) displayed mild mitochondrial swelling but maintained overall myofibril integrity. Group D (HF) demonstrated moderate mitochondrial swelling and aggregation with partial disruption of myofibril arrangement. Group E (Control) exhibited normal myocardial ultrastructure with well-organized sarcomeres, intact mitochondria with clear cristae, and regular Z-line arrangement (Figure 10).

### 3.12 Light microscopy pathological findings

**FIGURE 11:**
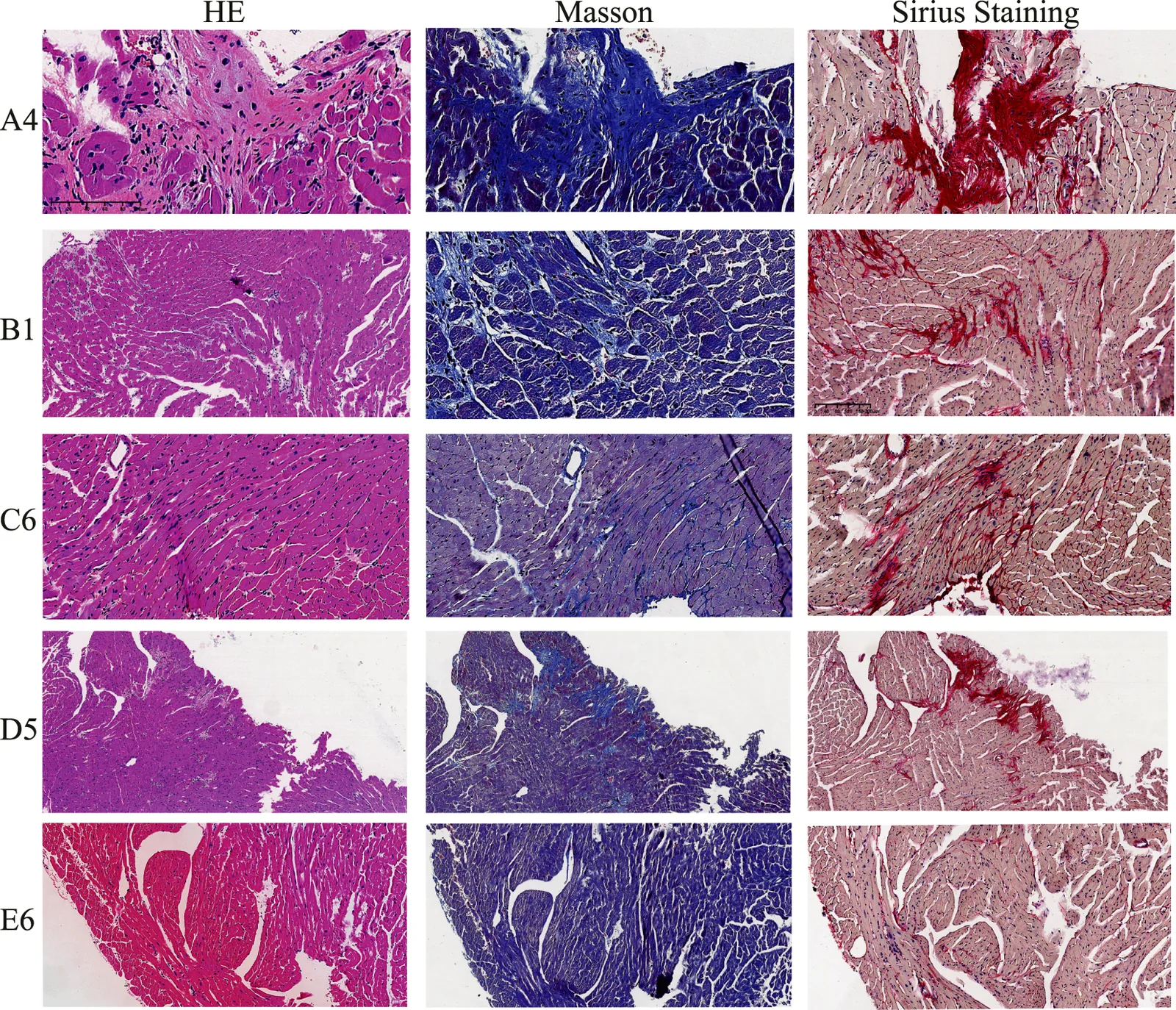
Histopathological analysis of myocardial tissue across experimental groups. Representative histological images of myocardial tissue stained with three different methods (×400 magnification). HE staining (upper panel): Shows myocardial cell morphology and inflammatory cell infiltration. Masson trichrome staining (middle panel): Blue areas indicate collagen fiber deposition, reflecting the degree of myocardial fibrosis. Sirius red staining (lower panel): Red areas represent collagen fibers, providing enhanced visualization of myocardial fibrosis. A4 (LHF): Heart failure with low-protein diet showing the most severe myocardial damage and extensive fibrosis. B1 ( LHF + Pro): Heart failure with low-protein diet plus probiotics showing reduced fibrosis. C6 (HF + Pro): Heart failure with normal diet plus probiotics showing moderate fibrosis. D5 (HF): Heart failure with normal diet showing moderate to severe fibrosis. E6 (Control): Control group displaying normal myocardial structure with minimal collagen deposition. Scale bar = 50 μm.

Histopathological examination revealed varying degrees of myocardial damage and fibrosis across the experimental groups. HE staining showed that Group A4 (LHF) exhibited the most severe myocardial injury with marked inflammatory cell infiltration, disrupted myocardial fiber arrangement, and myocyte degeneration. Group E6 (Control) displayed normal myocardial architecture with well-organized cardiomyocytes and minimal inflammatory infiltration. Masson trichrome staining demonstrated significant interstitial fibrosis (blue collagen deposition) in heart failure groups, with Group A4 showing the most extensive collagen accumulation. Group E6 showed minimal collagen content, indicating preserved myocardial structure. Probiotic intervention groups (B1 and C6) exhibited reduced fibrosis compared to their respective non-probiotic counterparts. Sirius red staining confirmed the Masson staining results, revealing pronounced collagen deposition (red staining) in Group A4, while Group E6 maintained normal collagen distribution. The collagen volume fraction was significantly elevated in the low-protein diet group and partially ameliorated by probiotic supplementation (Figure 11).

## 4 Discussion

Heart failure remains a significant global health burden with complex pathophysiological mechanisms (Groenewegen et al., 2020; Virani et al., 2021). The present study provides multi-level evidence, spanning mRNA profiling, protein expression, microbiome composition, and cardiac phenotype, to examine the role of the NLRP3 inflammasome pathway in heart failure. Our principal finding is that NLRP3 inflammasome proteins (NLRP3, TXNIP, ASC, IL-1β, IL-18) are upregulated in failing myocardium at the protein level, with protein expression levels correlating with functional cardiac impairment, microbiota dysbiosis severity, and histopathological injury grade. At the mRNA level, directionally consistent but statistically non-significant trends were observed, reflecting the limited sample size of the PCR analyses (n = 3 per group); these PCR findings should not be interpreted as confirmatory evidence of transcriptional upregulation. Dietary protein restriction amplified protein-level inflammasome expression while probiotic intervention was associated with its attenuation, suggesting that nutritional and microbiota-targeted strategies may modulate the NLRP3 pathway. These findings are exploratory and hypothesis-generating; causal inferences await confirmation in adequately powered studies.

The NLRP3 inflammasome has emerged as a central mediator of inflammatory responses in cardiovascular diseases (Butts et al., 2015; Wang et al., 2023). Our Western blot results demonstrated significantly elevated expression of NLRP3, ASC, IL-1β, and IL-18 proteins in all heart failure groups compared to controls, consistent with previous studies showing NLRP3 inflammasome activation in failing hearts (Mezzaroma et al., 2011). This activation initiates a cascade of inflammatory events that contribute to myocardial injury, fibrosis, and ventricular remodeling (Heymans et al., 2009).

The NLRP3 inflammasome functions as a molecular platform that senses danger-associated molecular patterns (DAMPs) and pathogen-associated molecular patterns (PAMPs) in the stressed myocardium (Schroder and Tschopp, 2010). Upon activation, it facilitates caspase-1-mediated processing of pro-IL-1β and pro-IL-18 into their mature, bioactive forms (Dinarello, 2009). These cytokines then amplify inflammatory responses by recruiting immune cells, promoting fibroblast activation, and inducing cardiomyocyte death through both apoptotic and pyroptotic pathways (Man et al., 2017). Our histopathological findings of increased inflammatory cell infiltration and myocardial fibrosis in heart failure groups support this mechanistic framework.

A particularly noteworthy finding in our study was the directional trend toward upregulation of TXNIP among all inflammasome components in the low-protein diet cohort, based on protein-level Western blot data; the mRNA-level findings, though directionally consistent, did not reach statistical significance (P = 0.424). TXNIP serves as a crucial molecular switch linking oxidative stress with NLRP3 inflammasome activation at the gene level (Patwari et al., 2006). The positive correlation between TXNIP protein expression and myocardial collagen volume fraction (r = +0.79) identified in our integrated analysis suggests a potential association between oxidative stress-sensitive TXNIP expression and fibrogenic remodeling in the failing heart. Whether this reflects a direct causal role of TXNIP in driving fibrosis or a shared upstream regulatory mechanism cannot be established from the current correlational design; direct mechanistic evidence such as TXNIP-specific siRNA silencing or knockout models would be required to establish causation. These findings nevertheless support TXNIP as a candidate target warranting further mechanistic investigation in heart failure. Under physiological conditions, TXNIP binds to thioredoxin (TRX), maintaining the NLRP3 inflammasome in an inactive state. However, during oxidative stress, reactive oxygen species (ROS) production causes TXNIP to dissociate from TRX, allowing free TXNIP to bind directly to NLRP3 and trigger inflammasome assembly (Wang and Yoshioka, 2017).

The electron microscopy findings in our study revealed significant mitochondrial damage, including mitochondrial swelling and cristae disruption, particularly in the low-protein diet group. These ultrastructural changes indicate mitochondrial dysfunction, which is both a consequence and a driver of oxidative stress (Doenst et al., 2013). Dysfunctional mitochondria generate excessive ROS, creating a vicious cycle of TXNIP activation, NLRP3 inflammasome assembly, and further cellular damage. This mechanism may explain why the low-protein diet group exhibited the most severe NLRP3 pathway activation and myocardial injury.

Our 16S rRNA sequencing results revealed substantial alterations in gut microbiota composition in heart failure rats, characterized by reduced microbial diversity and shifts in bacterial phyla distribution. These findings align with accumulating evidence that heart failure is associated with gut dysbiosis (Sun et al., 2021). The ‘gut hypothesis of heart failure’ proposes that decreased cardiac output leads to intestinal hypoperfusion and edema, resulting in impaired intestinal barrier function and bacterial translocation (Sandek et al., 2014).

The gut microbiota influences cardiovascular health through multiple mechanisms, including production of bioactive metabolites, modulation of immune responses, and regulation of systemic inflammation (Jie et al., 2017). In our study, TMAO levels showed a paradoxical pattern: decreased in HF and HF + Pro groups yet markedly elevated in the LHF group. Several explanations merit consideration. First, the low-protein diet in the LHF group, while restricting total protein, may have provided relatively higher dietary choline and carnitine proportions, increasing substrate availability for microbial TMAO synthesis. Second, standard heart failure may suppress hepatic FMO3 expression — reducing TMAO conversion — an effect that could be overridden by the excess substrate supply in LHF animals. Third, impaired renal clearance in the most hemodynamically compromised animals may contribute to TMAO accumulation. We acknowledge that without direct FMO3 expression data or renal function markers in this study, a definitive mechanistic explanation cannot be provided. Accordingly, we have moderated TMAO’s role from a confirmed axis component to a metabolite warranting further investigation. The gut dysbiosis pattern we observed, with enrichment of potentially pathogenic bacteria and depletion of beneficial species, nevertheless remains consistent with previous reports in heart failure patients (Kamo et al., 2017).

The bidirectional relationship between the gut and heart creates a pathophysiological feedback loop. Heart failure induces gut dysbiosis, which in turn produces pro-inflammatory metabolites and allows bacterial product translocation, further exacerbating cardiac inflammation and dysfunction (Tang et al., 2019). This gut-heart axis represents a promising therapeutic target for heart failure management. It should be noted that the PCA and hierarchical clustering analyses presented in this study are intended as descriptive tools to visualize overall trends in the multidimensional dataset. Given the limited number of animals per group (n = 4–6), these analyses lack the statistical stability expected of confirmatory analyses, and the clustering patterns observed should not be interpreted as definitive evidence of biologically distinct states. Replication in larger, adequately powered cohorts is necessary before such conclusions can be drawn.

A striking observation in our study was that low-protein diet significantly worsened heart failure outcomes, as evidenced by the most severe NLRP3 inflammasome activation, myocardial fibrosis, and ultrastructural damage in this group. This finding challenges the notion that protein restriction might be universally beneficial in cardiovascular disease. Our results suggest that inadequate protein intake may have detrimental effects on cardiac remodeling and function in heart failure.

The aggravated phenotype in the low-protein diet group may be attributed to several factors. First, insufficient protein intake can lead to malnutrition and cachexia, conditions commonly observed in advanced heart failure that independently predict poor outcomes (Anker et al., 1997). Second, protein deficiency may impair synthesis of antioxidant enzymes and repair proteins, rendering cardiomyocytes more vulnerable to oxidative injury. These findings underscore the importance of adequate nutritional support in heart failure patients and suggest that indiscriminate protein restriction should be avoided.

The probiotic intervention groups in our study demonstrated attenuated NLRP3 inflammasome activation, reduced myocardial fibrosis, and improved ultrastructural preservation compared to their respective non-probiotic counterparts. These findings support a growing body of evidence suggesting beneficial effects of probiotics in cardiovascular diseases (Gan et al., 2014).

First, probiotics can restore gut microbiota balance, promoting the growth of beneficial bacteria while suppressing pathogenic species. Our LEfSe analysis showed that probiotic-supplemented groups had enrichment of *Lactobacillus* and Bifidobacterium species, which are known to produce anti-inflammatory compounds and strengthen intestinal barrier function. Second, probiotics may reduce systemic inflammation by decreasing bacterial translocation and modulating immune cell function.

Third, certain probiotic strains can metabolize dietary precursors in ways that reduce production of harmful metabolites like TMAO (Tripolt et al., 2015). By competing with TMAO-producing bacteria or directly metabolizing choline and carnitine through alternative pathways, probiotics may mitigate the cardiovascular toxicity of gut-derived metabolites.

Our findings have several important clinical implications. First, the NLRP3 inflammasome represents a potential therapeutic target for heart failure treatment. Several NLRP3 inhibitors are currently in development, and our results support their potential utility in attenuating cardiac inflammation and remodeling. Second, maintenance of adequate protein nutrition should be emphasized in heart failure management, as protein restriction may inadvertently worsen outcomes.

Third, probiotic supplementation emerges as a promising adjunctive therapy for heart failure. The relative safety and accessibility of probiotics make them attractive candidates for clinical translation. Future clinical trials should investigate optimal probiotic strains, dosing regimens, and patient selection criteria for cardiovascular applications.

Several limitations of this study warrant acknowledgment. First, the group sizes were small (n = 3–6 per group) and no formal *a priori* power calculation was performed. The PCR analyses with n = 3 per group were underpowered to detect statistically significant transcriptional differences, and the reported correlation coefficients should be interpreted cautiously as they are susceptible to inflation at low sample sizes. All gene expression findings should be regarded as exploratory and hypothesis-generating rather than confirmatory. Second, this was an animal model study and findings may not directly translate to human heart failure. Third, the study design is correlational; the observed associations between probiotic intervention and reduced NLRP3 pathway expression cannot establish transcriptional causation without direct mechanistic evidence such as chromatin immunoprecipitation or gene silencing experiments. Fourth, the absence of a heat-killed probiotic or sham-gavage control group means that gavage stress cannot be fully excluded as a confounding variable in the probiotic arms. Fifth, we did not perform comprehensive metabolomic profiling beyond TMAO measurement, and FMO3 expression and renal function markers were not assessed, limiting mechanistic interpretation of the paradoxical TMAO findings. Sixth, the echocardiographic assessment was restricted to LVEDD, LVESD, LVEF, and LVFS, providing only a partial characterization of cardiac remodeling. A more comprehensive assessment including tissue Doppler imaging, myocardial performance index, and diastolic function parameters would be valuable in future studies. Seventh, echocardiographic measurements were performed by a single operator who was not blinded to group allocation, which may introduce measurement bias. Future studies should employ adequately powered cohorts, multi-omics approaches, and direct mechanistic tools to comprehensively characterize gut-heart axis alterations in heart failure.

## 5 Conclusion

In summary, our integrated molecular study demonstrates that protein expression of the NLRP3 inflammasome pathway (NLRP3, TXNIP, ASC, IL-1β, IL-18) is upregulated in heart failure and correlates with diet-dependent cardiac inflammation, fibrosis, and dysfunction. PCR-based mRNA analyses showed directionally consistent trends that did not reach statistical significance in this sample size (n = 3 per group), and should be considered exploratory. The correlations between inflammasome protein expression and cardiac functional indices, gut microbiota diversity, and serum biomarkers suggest these proteins as candidate disease markers. Low-protein diet amplified the NLRP3 pathway, possibly in association with TXNIP-related oxidative stress signaling, though direct mechanistic evidence is lacking; probiotic supplementation was associated with reduced pathway activation through microbiome modulation. These findings are hypothesis-generating and support the further investigation of the NLRP3-TXNIP axis and microbiota-targeted interventions in heart failure. Future studies should employ adequately powered cohorts, single-cell RNA sequencing, and chromatin immunoprecipitation to resolve causal gene regulatory mechanisms within the failing myocardium.

## Data Availability

The 16S rRNA gene sequencing data presented in this study are deposited in the NCBI Sequence Read Archive under BioProject accession number PRJNA1266395. The remaining data supporting the conclusions of this article are included in the article; further inquiries can be directed to the corresponding author.
